# Immediate relief of herniated lumbar disc-related sciatica by ankle acupuncture: A study protocol for a randomized controlled clinical trial

**DOI:** 10.1097/MD.0000000000009191

**Published:** 2017-12-22

**Authors:** Anfeng Xiang, Mingshu Xu, Yan Liang, Jinzi Wei, Sheng Liu

**Affiliations:** a Shanghai University of Traditional Chinese Medicine, Shanghai, China; b Shanghai Yueyang Hospital affiliated to Shanghai University of Traditional Chinese Medicine, China.

**Keywords:** ankle acupuncture, herniated intervertebral disc, immediate relief, sciatica

## Abstract

**Background:**

Around 90% of sciatica cases are due to a herniated intervertebral disc in the lumbar region. Ankle acupuncture (AA) has been reported to be effective in the treatment of acute nonspecific low back pain. This study aims to evaluate the efficacy of a single session of ankle acupuncture for disc-related sciatica.

**Method:**

This will be a double-blinded, randomized controlled clinical trial. Patients diagnosed with disc-related sciatica will be randomly divided into 3 parallel groups. The treatment group (n = 30) will receive ankle acupuncture. The 2 control groups will either undergo traditional needle manipulation (n = 30) or sham acupuncture (n = 30) at the same point as the treatment group. The primary outcome will be pain intensity on a visual analog scale (VAS). The secondary outcomes will be paresthesia intensity on a VAS and the Abbreviated Acceptability Rating Profile (AAPR). The success of blinding will be evaluated, and the needle-induced sensation and adverse events will be recorded. All outcomes will be evaluated before, during, and after the treatment.

**Discussion:**

This study will determine the immediate effect and specificity of ankle acupuncture for the treatment of disc-related sciatica. We anticipate that ankle acupuncture might be more effective than traditional needle manipulation or sham acupuncture.

**Trial registration:**

ChiCTR-IPR-15007127 (http://www.chictr.org.cn/showprojen.aspx?proj=11989)

## 1 Introduction

Sciatica is characterized by sciatic nerve pain (pain that radiates from the low back to below the knee), paresthesia (mostly numbness and tingling), and muscle weakness in the affected leg or foot.^[2]^ These symptoms can lead to motor disabilities^[1]^ and mental disorders such as depression and anxiety.^[3]^ The prevalence of sciatica ranges from 1.2% to 43%.^[4]^ The most common cause of sciatica is herniation of the nucleus pulposus. Indeed, about 90% of sciatica cases are due to a herniated disc involving nerve root compression. Other possible causes include a narrow lumbar canal, foraminal stenosis, tumors, and cysts.^[5]^ Herniated lumbar disc-related sciatica is one of the most common conditions managed in primary medical care and a significant cause of absence from work and early retirement. Patients, families, and the society at large all carry part of the burden.^[6]^

Sciatica resolves without treatment in the majority of cases. However, many patients endure substantial pain and disability.^[7,8]^ Medication and physical therapy are used as the initial treatment options for pain control. However, there is little evidence for the efficacy of pain medication.^[9]^ Several systematic reviews have analyzed the efficacy and safety of various drugs such as opioids and steroids, but the validity of the studies is limited.^[10–12]^ Furthermore, the use of pain medication has been associated with adverse effects such as sedation, dizziness, ataxia, and nausea.^[13]^ Similarly, there is little evidence for the efficacy of invasive surgeries such as lumbar discectomy and epidural steroid injections (ESIs).^[8,14,15]^

Acupuncture and electroacupuncture (EA) have been applied for the treatment of sciatica since the early 1990s,^[16–18]^ but results of studies on their efficacy have been inconsistent. According to the theory of Traditional Chinese Medicine (TCM), acupuncture needles are inserted into the body, and sufficient manual needling manipulation (e.g., lifting-thrusting and twisting-rotating) of the inserted needles elicits a composite of sensations (the response of the characteristic needle-manipulation sensation is termed *deqi*, which generally manifests as numbness, heaviness, distention, and soreness), which is believed to be an indispensable component in achieving a therapeutic effect. It is noteworthy that in clinical practice, many patients are afraid of the *deqi* sensation. Fear of needle-manipulation sensation is an important reason as to why patients choose to forego acupuncture treatment.

Ankle acupuncture (AA) is a type of subcutaneous acupuncture that has been developed in the 1970s.^[19]^ Compared with traditional acupuncture manipulation, AA penetrates the skin and inserts the needles shallowly in the subcutaneous tissue above the ankle, with no characteristic needle-manipulation sensation. Patients are more likely to accept AA due to the absence of needle-manipulation sensation. In addition, as it requires only the insertion of a single needle with no further manual manipulation, it can be easily taught to medical staff without much knowledge about acupuncture. Accumulating evidence from Chinese literature sources reveals that AA has similar therapeutic effects as traditional acupuncture in the treatment of pain.^[20]^ Some studies have reported the efficacy of AA for the treatment of sciatica.^[21,22]^ However, these studies were hampered by insufficient reporting methods of randomization and allocation concealment, improper blinding, and inadequate strength of the inference. To date, no appropriately powered randomized controlled trials (RCTs) of AA for sciatic pain associated with a herniated lumbar disc have been conducted.

Time-dependent characteristics of acupuncture might be one of the most important factors in the assessment of its effects in the relief of pain. Based on the time-dependent characteristics of acupuncture, the effects of acupuncture are generally classified as either immediate (after the end of the first treatment session) or cumulative (after repeated acupuncture stimulation).^[23,24]^ So far, most clinical trials and systematic reviews assessing the role of acupuncture have focused on the cumulative effects of repeated acupuncture stimulation. However, the immediate effects could have clinical significance and methodological advantages. Immediate pain reduction might motivate the patient to continue treatment and facilitate further physiotherapy. Patients who receive little benefit or no immediate analgesic effect following the first treatment might be expected to be less likely to gain benefit from repeated stimulation. Notably, for many patients with acute pain such as that resulting from surgery or childbirth, the reported analgesic effects of acupuncture are usually immediate. Furthermore, the results of some functional magnetic resonance imaging (fMRI) studies have suggested that the immediate and cumulative effects of acupuncture treatment elicit different temporal neural responses in a wide range of brain networks,^[25,26]^ which could explain the underlying specific mechanisms of the immediate effects of acupuncture.

Therefore, we propose an RCT to evaluate the immediate effect of AA for sciatic pain associated with a herniated lumbar disc. The results of our clinical trial might guide better rational and selective use of acupuncture as a treatment for sciatica in the future.

## 2 Methods

### 2.1 Trial design

**Figure 1:**
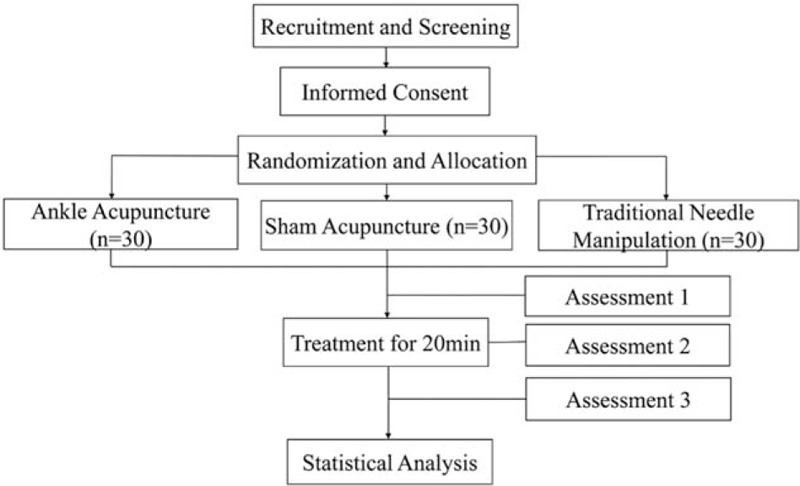
Participant flow diagram.

**Figure 2:**
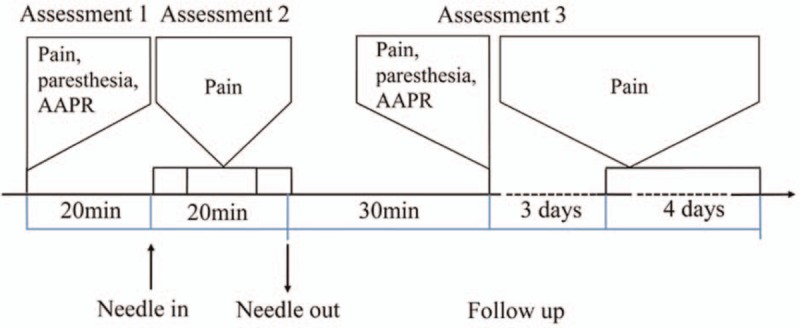
The trial schedule. AAPR. AAPR = Abbreviated Acceptability Rating Profile.

The study is designed to be a randomized, three-arm, parallel-group, controlled trial to evaluate the immediate efficacy, safety, and acceptability of AA therapy for sciatic pain due to a herniated lumbar disc. The three arms are AA, sham AA, and traditional needle manipulation (TNM). The clinical trial is patient/assessor-blinded, thus adhering strictly to SPIRIT (Standard Protocol Items for Randomized Trials) guidelines.^[27]^ The flowchart of the study protocol is shown in Figure 1 and the trial schedule is shown in Figure 2.

### 2.2 Ethics and registration

The study protocol (version 3.0, informed consent form included) is in line with the Declaration of Helsinki and has been approved by the Institutional Review Board (IRB) of Shanghai Yueyang Hospital affiliated to Shanghai University of Traditional Chinese Medicine (reference: 201517). This trial has been registered with the Chinese Clinical Trial Registry (ChiCTR-IPR-15007127, URL: http://www.chictr.org.cn/showprojen.aspx?proj=11989). Before randomization, all patients will be requested to sign a written informed consent, and they will be absolutely free to choose whether or not to continue the trial at any time.

## 3 Participants

### 3.1 Inclusion criteria

Patients will be included if they meet all of the following criteria: (1) age between 18 and 55 years (either sex); (2) primary complaint of unilateral leg pain radiating below the knee; (3) ipsilateral lumbar disc herniation at the corresponding level verified by MRI or computed tomography^[28]^; (4) duration of pain < 5 years; (5) pain intensity score of 40 mm or more on a 100-mm visual analog scale (VAS); (6) willingness and ability to accept needle-acupuncture intervention and to comply with the requirements of the study protocol; and (7) signed informed consent.

### 3.2 Exclusion criteria

Patients with any of the following conditions will be excluded: (1) leg pain originating from the upper lumbar column (L1–L3); (2) history of spine trauma or spine surgery; (3) history of stroke, heart diseases or severe hypertension, any endocrine diseases such as hyperthyroidism, and severe infection; and (4) other therapies, especially analgesics, in the past 7 days.

### 3.3 Recruitment and screening procedures

Patients will be recruited at Shanghai Yueyang Hospital affiliated to Shanghai University of Traditional Chinese Medicine, with a target sample size of 90 subjects. Two strategies will be adopted to recruit participants with sciatica. One is to recruit participants in outpatient clinics. The other is to share electronic posters (containing brief introductions about the population needed, the free acupuncture treatment offered to eligible participants, and the contact information of the researcher) via the Internet.

The initial phone screening, which will involve recording the age and course of low back pain will determine potential eligibility, and then screening forms will be completed by interested participants via a face-to-face interview (diagnosis by a physician and assessment by a researcher) at the hospital sites. Only those interested participants who meet the eligibility criteria will be scheduled to sign written informed consent.

### 3.4 Randomization/allocation

Only those participants who have signed informed consent and go through the screening will be assigned randomly to one of the three interventions in a ratio of 1:1:1. Randomization number will be generated using a computerized number generator through the stratified block randomization method of the Statistical Analysis System (SAS) package (V.9.1.3; SAS Institute Inc., Cary, North Carolina) by a researcher not involved in this trial. The random numbers will be stored in sealed envelopes for random allocation until an investigator opens each envelope closely before intervention.

### 3.5 Blinding

AA inserts needles into the subcutaneous tissue with no needle-manipulation sensation; sham AA is non-penetrating with a slight prick on the skin; and TNM involves penetration with no needle manipulation. Therefore, it is possible to blind the patients to the treatment. All participants will be told that they will receive an innovative type of acupuncture and that they may not feel any sensation characteristic of traditional acupuncture except for minor pain caused by puncturing through the skin. The participants will be treated separately using the same equipment (disposable sterile steel needles retained for a total of 20 minutes, 75% alcohol to disinfect the skin) and instructed during intervention without any implying words. Outcome assessors and data analysts will be masked to the intervention allocation throughout the study.^[29]^ Although acupuncturists will not be blinded to group allocation, they will not be involved in the outcome assessments or data analyses.

## 4 Interventions

**Figure 3:**
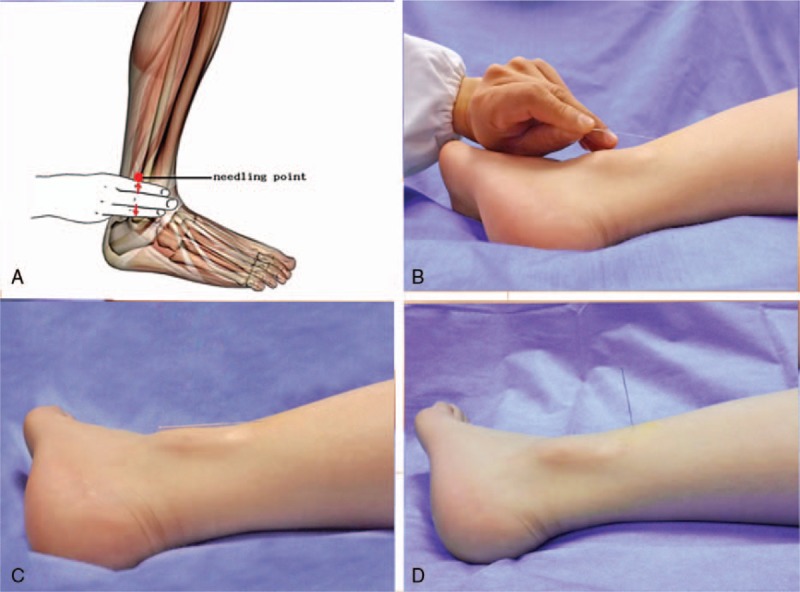
Photograph of the ankle acupuncture procedures and placement of acupuncture needles. The needling point at the ankle zone 5 of the affected leg. (A) The needle manipulation (Ankle acupuncture group, B and C). The needle manipulation (traditional needle manipulation group, D).

The patients will be guided to lay supine on a couch with their affected leg exposed. As shown in Figure 3A, the needling point will be at the ankle zone 5 of the affected leg. This point is located three fingers above the lateral malleolus and posterior margin.^[19]^

One certified acupuncturist with more than 5 years of clinical practice at each site will perform AA, sham AA, and TNM. The needle will be retained for 20 minutes and no manual needling manipulation will be conducted before the needle is withdrawn.

### 4.1 AA group

After sterilizing with 75% ethanol at and around the needling point, a disposable sterile acupuncture needle (0.35 mm × 40 mm; Tianxie Brand, Beijing, China) will be used. The acupuncturist will hold the needle and swiftly insert it into the skin at the target point with a 30° angle to the skin. Then, the needle will be slowly advanced with a 0° angle to the skin until about 3/4 of the needle enters the subcutaneous tissue. The patients will not feel any *deqi* during this process if the AA is performed correctly. Then, the needle will be retained in the subcutaneous tissue for 20 minutes with the needle handle fixed to the skin with an adhesive tape (Fig. 3B and C).

### 4.2 Sham AA group

A disposable sterile acupuncture needle (0.35 mm × 13 mm; Tianxie Brand) will be blunted before intervention so that it cannot be inserted into the skin easily. The skin will be sterilized as in the AA group. The acupuncturist will hold the blunt needle and prick the skin for 3 seconds, after which the needle will be pushed down to the skin. The needle will be retained for 20 minutes with the needle handle fixed to the skin with an adhesive tape.

### 4.3 TNM group

After sterilizing with 75% ethanol, a disposable sterile acupuncture needle (0.35 mm × 13 mm; Tianxie Brand) will be used. The acupuncturist will hold the needle perpendicularly to the skin and swiftly insert to a depth of 2–3 cm into the skin at the target point. Then, the needle will be retained for 20 minutes without any movement (Fig. 3D).

## 5 Outcome assessment

### 5.1 Primary outcome measures

The primary outcome will be pain intensity on a VAS,^[30]^ with the primary endpoint at 0 minutes (immediately after treatment). The secondary time point will be at 2 hours after acupuncture treatment, and an area-under-curve analysis will be performed, including baseline, 15 minutes, 30 minutes, 3 days, and 7 days. We chose VAS because it is commonly used to measure pain intensity.^[31]^ The patients will be guided to mark a point on a 0 to 100 mm VAS, in which 100 (at the right end) indicates maximum pain and 0 (at the left end) indicates absolutely no pain.

### 5.2 Secondary outcome measures

Given that paresthesia (e.g., numbness and tingling) is the second most common symptom in sciatica,^[32]^ the sensation will be measured as one of the secondary outcomes. Paresthesia intensity will be assessed using a 100-mm VAS, in which “0” indicates the absence of numbness and tingling and “100” indicates unbearable numbness and tingling.

The sensation of needling is one of the significant outcomes on reliability of the operation. The sensation of needling will be assessed using self-report measures after the needle is withdrawn. The participants will be asked to report any *deqi* sensation such as sourness, numbness, heaviness, warmness, and pain, among others.

Acceptability ratings will be obtained using the Abbreviated Acceptability Rating Profile (AAPR).^[33]^ In AARP, higher scores indicate greater acceptability. The reliability and validity of the Chinese version of AAPR are satisfactory.^[34]^

Any additional treatments received by participants will be asked to report at each follow-up.

Any severe adverse events such as bleeding, hematoma, or fainting during the acupuncture process will be observed and dealt with immediately. The needle will be removed once any of the above-mentioned severe events occur.

### 5.3 Data collection and management

To evaluate the effect of AA, data will be collected at baseline, upon completion of the intervention, and at short-term follow-up (days 3 and 7 after acupuncture). The data items and the corresponding timeline are summarized in Table 1. A case report form (CRF) has been designed to include the variables of interest. To increase the objectiveness of subsequent statistical analysis, the information obtained by CRF will be transcribed to an electronic database by 2 independent researchers; any disagreement will be solved by discussion. To protect confidentiality of participants, initials will be recorded in CRFs, and all CRFs will be stored in a locked cabinet in Shanghai University of Traditional Chinese Medicine, and will have a unique identification number. The access to the database will be restricted to the researchers in this study team.

**Table 1:**
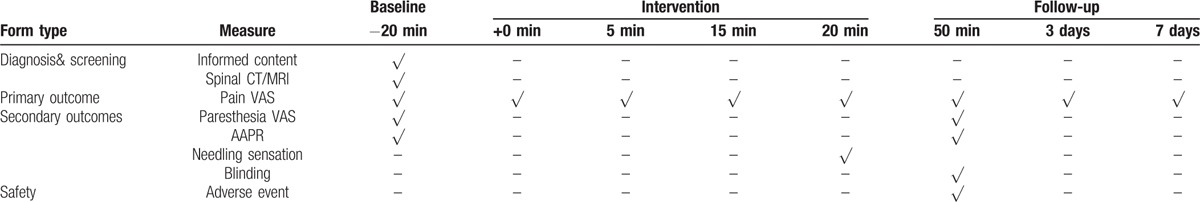
The schedule of follow-up.

## 6 Statistical methods

### 6.1 Sample size calculation

A repeated measures design was employed for sample size calculation with GLIMMPSE software (URL: *http://glimmpse.samplesizeshop.org/*) based on the study design and the results of our previous clinical trial.^[35]^ Pain will be measured on a VAS 8 times per patient. When a two-tailed test with a significance level of 5% and a test power of 90% was applied, the number of participants required per group was 26. Therefore, a target sample size of 90 participants is required, assuming a 15% dropout rate. Each group will include 30 initial participants.

### 6.2 Statistical analysis

The Statistical Package for the Social Sciences (SPSS version 21.0) will be used for all statistical analyses. Baseline demographic variables and clinical characteristics will be compared across groups using one-way analysis of variance (ANOVA) for continuous variables and Pearson's χ^2^ tests for categorical variables. The differences in pain VAS scores and paresthesia VAS scores between the AA, sham AA, and TNM groups will be analyzed using repeated measures ANOVA with time as a repeated within-subject factor and group as a between-subject factor. Fisher's least significant difference post-hoc test will be used to compare the effects of the treatments at each time point. Sensation of needling and acceptability ratings (AAPR) will be compared across groups using one-way ANOVA. Pearson's χ^2^ tests will be used to examine the differences between groups. All tests will be two-tailed, with the significance level set at 0.05. Per-protocol (PP) analysis and intention-to-treat (ITT) analysis of the results will be conducted.

## 7 Discussion

AA has been recognized as a readily available and valuable means of health care. Different from traditional acupuncture, AA penetrates the skin and inserts the needles shallowly in the subcutaneous tissue above the ankle, with no characteristic needle-manipulation sensation. Therefore, the procedure is not only effective, but also requires only simple equipment and is inexpensive, relatively safe, convenient, and quick. However, the practice of AA is still mainly based on personal experience. Further clinical research on AA will provide additional evidence to confirm its effectiveness, thereby enhancing its acceptance and utilization. The purpose of this study is to evaluate the immediate effect of ankle acupuncture for sciatica associated with a herniated lumbar disc. We propose the following specific aims: (1) to determine if patients receiving AA show significant improvements in pain as measured by VAS compared to control patients; and (2) to determine the safety and acceptability of AA therapy by assessing its possible adverse effects and acceptability ratings after one session of treatment. One of the strengths of our study is that it is designed to be a feasible, comparative effectiveness trial design that is similar to common clinical situations. The results of this study will determine if AA is an effective therapy for sciatica due to a herniated lumbar disc.

There is considerable evidence to show that acupuncture treatment produces pain relief for durations that outlast the period of stimulation.^[36]^ These prolonged effects after cessation of acupuncture stimulation may undergo 2 phases: (1) sudden, dramatic relief of pain for several hours after stimulation, and (2) gradual pain relief that returns days or weeks after a single treatment session.^[37]^ In this study, we will observe the duration of pain relief to provide information as to how long pain relief lasts after a single session of AA. We will set 2 follow-up time points: 3 and 7 days. Multiple time points will be used to reflect the time-effect of AA for sciatica associated with a herniated lumbar disc. Additional post-treatment measurements after single session of AA therapy will provide useful information to make definitive inferences about the clinical relevance and the underlying physiological mechanism of AA.

In this study, we will focus on the immediate effect of AA on sciatic pain associated with a herniated lumbar disc. The measurements will only be conducted for a single session of AA treatment. There are many methodological advantages. For example, natural remission of disease, regression to the mean in clinical studies, and other factors disturbing the internal validity of results should play a minor role compared with other study designs.^[38,39]^ However, it is notable that a single treatment is not adequate in patients suffering from chronic pain. From a clinical point of view, the cumulative effect of acupuncture has been considered as an important factor associated with its clinical efficacy.^[40]^ Given that cumulative effects of repeated acupuncture are not simply because of overlapping immediate analgesic effects,^[41,42]^ further rigorous, high-quality, RCTs comparing acupuncture with no treatment and sham acupuncture are required to evaluate the cumulative effects of repeated AA stimulation.

A major limitation of the present study is that the acupuncturist will not be blinded to the treatment. However, the patients will be blinded to the acupuncture treatments since they cannot distinguish true from sham AA. In addition, the treatment duration will be similar in the 3 groups, and pain will be evaluated by a clinician who is unaware of the treatment received, at a time when all needles have been withdrawn. In addition, the use of an appropriate control group is a critical issue in designing a high-quality clinical trial. It is difficult to provide scientifically robust sham treatments as controls because the specific mechanisms and causal pathways of AA are not known. For example, it is unknown whether there are neural pathways associated with both specific and nonspecific effects, such as those associated with self-healing. In this study, we set 2 control groups. In the sham AA group, the needle tip just pricks on the skin without penetrating. In the TNM group, the needle tip will be swiftly inserted into the skin at the target point at a 90-degree angle. Therefore, 2 control groups set in this study will prove the specificity of AA in the treatment of sciatic pain associated with a herniated lumbar disc.

In conclusion, this study will focus on the efficacy of AA for treating sciatic pain associated with a herniated lumbar disc. It will also be interesting to evaluate the safety and acceptability of AA treatment. We hope that this study provides useful information to advance the methodology of acupuncture trials and adds evidence for the effectiveness of AA for sciatic pain, with sufficient levels of acceptability and safety.

## 8 Authorship

AFX contributed to conception, design, and writing of the manuscript, MSX and YL contributed to conception and design, JZW contributed to conception and revising the manuscript critically and SL contributed to conception, writing of the manuscript, and revising the manuscript critically. All authors read and approved the final manuscript.

## Acknowledgments

We thank Dr Lixing Lao at the School of Chinese Medicine, Hong Kong University. We would like to thank Editage (www.editage.com) for English language editing.
